# THE EVOLUTION OF THE GERONTOLOGY PROGRAM AT ALFRED UNIVERSITY

**DOI:** 10.1093/geroni/igae098.1621

**Published:** 2024-12-31

**Authors:** Robert Maiden, Danielle Gagne

**Affiliations:** Alfred University, Alfred, New York, United States; Alfred University, Alfred, New York, United States

## Abstract

Alfred University instituted its major and minor in gerontology in 1980. At that time, it was one of two bachelor’s degrees in gerontology offered in the State of New York. It was established to meet the burgeoning need of competent service providers for older Americans. The need is clear. It is projected that there will be 89 million older Americans by 2050, which will increase twice as fast as the underage 50 population. Although there is growing need for competent service providers and myriad potential career opportunities for undergraduates, there has been a declining interest in the gerontology program. The gerontology program at Alfred University has not had any majors or minors for the past two years. Many reasons have been proffered for this decline, including that many students do not understand the word “gerontology” or have misconceptions and negative stereotypes regarding aging. To increase enrollment in gerontology, we propose two changes: 1) adding aging as a concentration to our psychology major with an interdisciplinary component; and 2) changing the name to “Aging Studies” or “Adult Development & Aging.” Moreover, students in this concentration can minor in business administration and become eligible for our 4 + 1 program, in which they can earn an MBA at Alfred University in one year post baccalaureate instead of the usual two.

